# Predictors of response to foot orthoses and corticosteroid injection for plantar heel pain

**DOI:** 10.1186/s13047-020-00428-6

**Published:** 2020-09-29

**Authors:** Glen A. Whittaker, Karl B. Landorf, Shannon E. Munteanu, Hylton B. Menz

**Affiliations:** 1 Discipline of Podiatry, School of Allied Health, Human Services and Sport, La Trobe University, Melbourne, Victoria 3086 Australia; 2 La Trobe Sport and Exercise Medicine Research Centre, La Trobe University, Melbourne, Victoria 3086 Australia

**Keywords:** Plantar fasciitis, Plantar heel pain, Orthotic devices, Foot orthoses, Corticosteroids, Linear regression, Fear-avoidance

## Abstract

**Background:**

Foot orthoses and corticosteroid injection are common interventions used for plantar heel pain, however few studies have investigated the variables that predict response to these interventions.

**Methods:**

Baseline variables (age, weight, height, body mass index (BMI), sex, education, foot pain, foot function, fear-avoidance beliefs and feelings, foot posture, weightbearing ankle dorsiflexion, plantar fascia thickness, and treatment preference) from a randomised trial in which participants received either foot orthoses or corticosteroid injection were used to predict change in the Foot Health Status Questionnaire foot pain and foot function subscales, and first-step pain measured using a visual analogue scale. Multivariable linear regression models were generated for different dependent variables (i.e. foot pain, foot function and first-step pain), for each intervention (i.e. foot orthoses and corticosteroid injection), and at different timepoints (i.e. weeks 4 and 12).

**Results:**

For foot orthoses at week 4, greater ankle dorsiflexion with the knee extended predicted reduction in foot pain (adjusted *R*^2^ = 0.16, *p* = 0.034), and lower fear-avoidance beliefs and feelings predicted improvement in foot function (adjusted *R*^2^ = 0.43, *p* = 0.001). At week 12, lower BMI predicted reduction in foot pain (adjusted *R*^2^ = 0.33, *p* < 0.001), improvement in foot function (adjusted *R*^2^ = 0.37, *p* < 0.001) and reduction in first-step pain (adjusted *R*^2^ 0.19, *p* = 0.011). For corticosteroid injection at week 4, there were no significant predictors for change in foot pain or foot function. At week 12, less weightbearing hours predicted reduction in foot pain (adjusted *R*^2^ = 0.25, *p* = 0.004) and lower baseline foot pain predicted improvement in foot function (adjusted *R*^2^ = 0.38, *p* < 0.001).

**Conclusions:**

People with plantar heel pain who use foot orthoses experience reduced foot pain if they have greater ankle dorsiflexion and lower BMI, while they experience improved foot function if they have lower fear-avoidance beliefs and lower BMI. People who receive a corticosteroid injection experience reduced foot pain if they weightbear for fewer hours, while they experience improved foot function if they have less baseline foot pain.

## Background

Plantar heel pain is a disabling foot condition that has a significant impact on health-related quality of life [1]. Prevalence estimates of plantar heel pain in the community range between 4 and 10% [2–4], while in athletes, prevalence estimates range between 5 and 18% [5]. Despite the relatively high prevalence of plantar heel pain, there is limited high-quality evidence to guide health professionals regarding the interventions that are most effective.

A recent randomised trial (the SOOTHE heel pain trial) aimed to clarify the effectiveness of two common interventions used to treat plantar heel pain, which were foot orthoses and corticosteroid injection [6]. The trial found that corticosteroid injection was more effective than foot orthoses in the short term (at week 4) and that foot orthoses were more effective than corticosteroid injection in the longer term (at week 12). The published article that presented the findings for this trial focused on the comparative effectiveness of these interventions, but it did not provide information about the variables that predict response to each intervention [6]. Understanding variables that predict response may help health professionals choose interventions that are most appropriate for their patients and assist researchers to design clinically relevant randomised trials.

To our knowledge, there is only one study that has evaluated variables that predict pain or function for foot orthoses and corticosteroid injection for plantar heel pain [7]. The authors evaluated variables to predict response to a combination of foot orthoses, calf stretches, new footwear, and ice massage for people with plantar heel pain. This study, which was a secondary analysis of a randomised trial, found that heel valgus in stance, and first-step pain greater than 7/10 on a visual analogue scale (VAS) were predictive of a *worse* response, while an inability to dorsiflex the ankle past 5 degrees with the knee extended using a non-weightbearing assessment was predictive of an *improved* response. There are no studies that have evaluated variables that predict change in pain or function for corticosteroid injection.

Given the available evidence, the aim of this study was to investigate which factors influence the response to foot orthoses and corticosteroid injection in people with plantar heel pain. Baseline variables (age, weight, height, body mass index (BMI), sex, education, foot pain, foot function, fear-avoidance beliefs and feelings, foot posture, ankle dorsiflexion, plantar fascia thickness, and treatment preference) were used to predict the change in foot pain, foot function, and first-step pain after 4 and 12 weeks.

## Methods

Data for this study were obtained from a published, assessor-blinded, randomised trial that evaluated the comparative effectiveness of foot orthoses and corticosteroid injection for plantar heel pain [6]. Detailed methods of the trial are available from the published protocol [8]. Participants were randomly allocated to receive either foot orthoses or a single ultrasound-guided corticosteroid injection and were followed up for 12 weeks. The trial was prospectively registered with the Australian New Zealand Clinical Trials Registry (registration ACTRN12615001266550). Ethical approval was obtained from the La Trobe University Human Ethics Committee (approval number 15–120) and all participants provided informed consent.

### Participants

Participants were included in the trial if they were over 18 years of age, had a diagnosis of plantar heel pain of at least 4 weeks duration [8, 9], and reported an average pain over the last 7 days of at least 30 mm on a 100 mm VAS. Participants were excluded if they had received any treatment in the past 4 weeks, had worn foot orthoses or received a corticosteroid injection in the heel in the last 6 months, had a history of surgery to the heel, or had a systemic medical condition such as an inflammatory disorder, connective tissue disease, or neurological disorder.

### Interventions

**Fig. 1:**
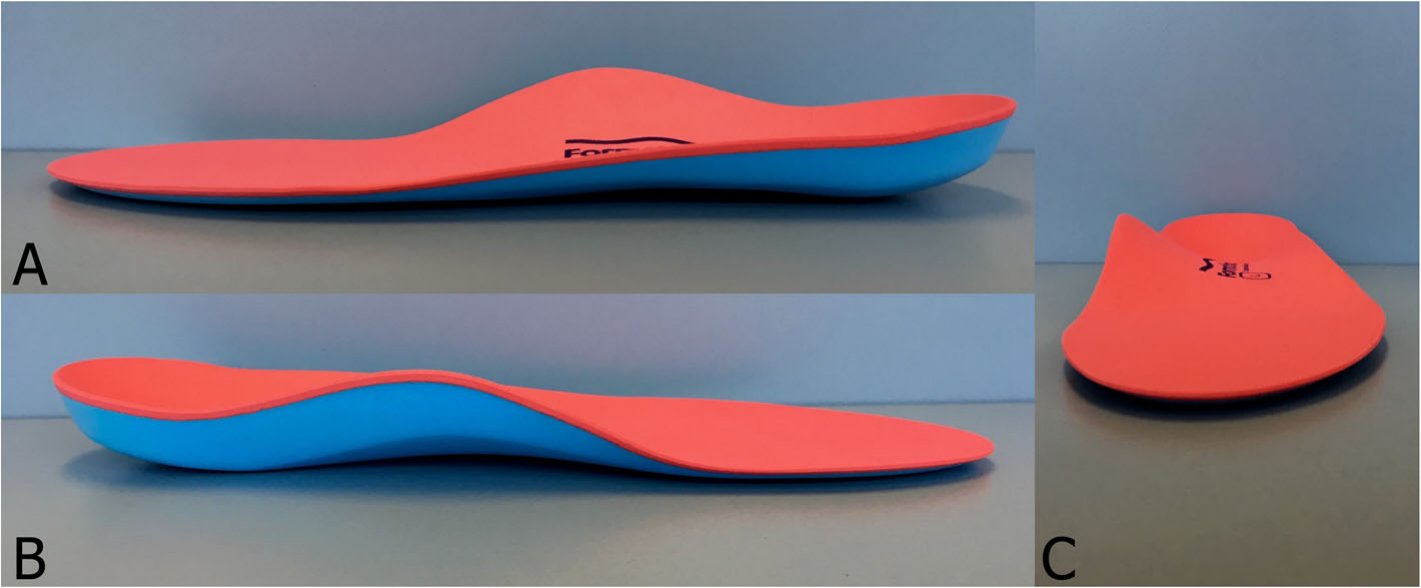
Formthotics full-length, dual-density, prefabricated foot orthosis. **a** View of the lateral orthosis, (**b**) view of the medial orthosis, and (**c**) view from the distal to the proximal end of the orthosis [8]

Participants randomised to the foot orthoses group received a pair of Formthotics™ prefabricated foot orthoses (Foot Science International, Christchurch, New Zealand). The Formthotics™ were full-length, dual-density devices manufactured from a soft polyethylene foam top layer (Shore A durometer 25) and a firm polyethylene foam base layer (Shore A durometer 50) (Fig. 1). The foot orthoses were fitted for each participant by a podiatrist who selected the appropriate size and heated the foot orthoses in the shoes with a device specifically designed for this purpose by Foot Science International. Each participant stood with the foot orthoses in their shoes to allow them to mould appropriately. Modifications were not made unless the participant experienced discomfort.

**Fig. 2:**
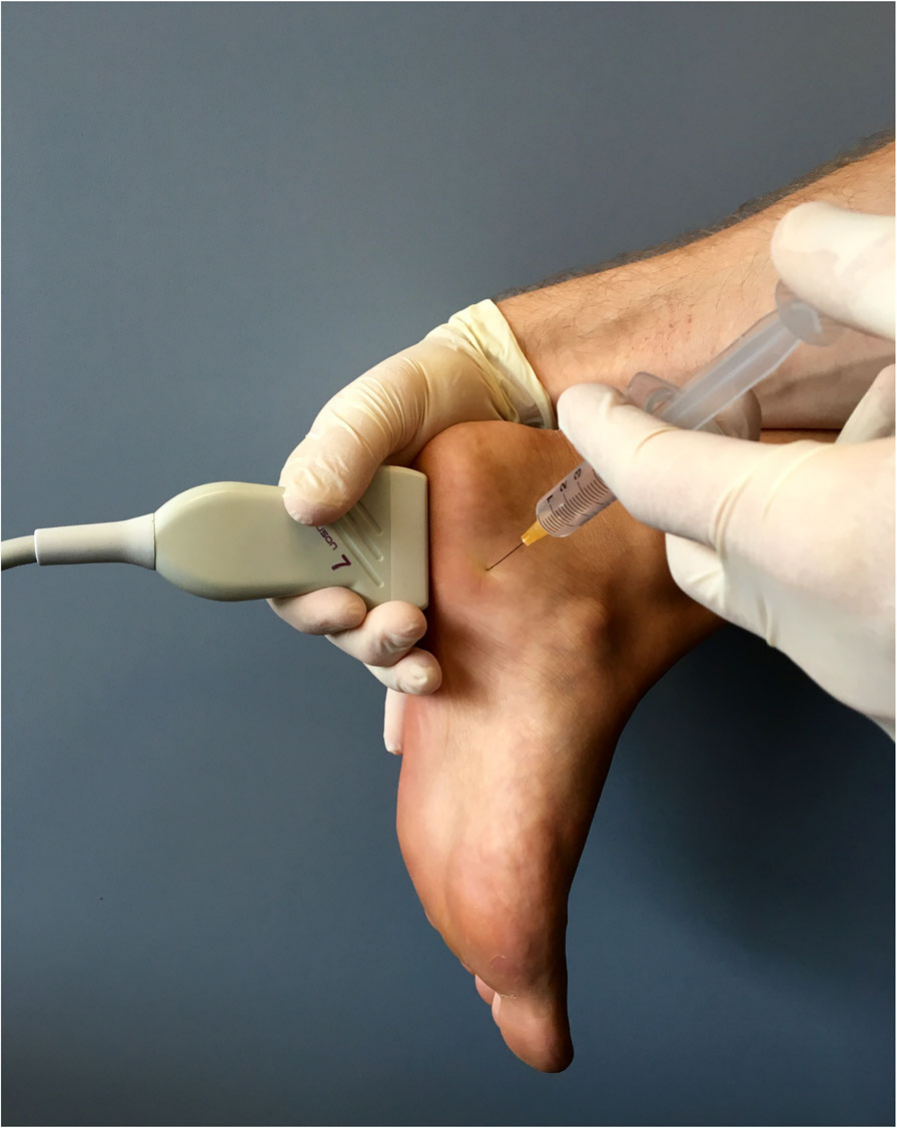
Technique used to administer the ultrasound-guided corticosteroid injection [8]

Participants randomised to the corticosteroid injection group received a single ultrasound-guided corticosteroid injection from a radiologist. Participants were placed in a prone position, with their feet hanging from the end of an examination table (Fig. 2). A 25 gauge needle was used to inject a solution containing 1 mL of a combination of betamethasone acetate and betamethasone sodium phosphate (Celestone® Chronodose®), and 1 mL of bupivacaine (Marcaine® 0.5%).

Participants in both groups received education about plantar heel pain, and were advised to carry out plantar fascia and calf stretches [8].

### Outcome measures

Outcome measures were obtained at baseline, week 4, week 8, and week 12. Several outcome measures were used for the randomised trial; the following outcome measures are those that are relevant to this study.

(i) Participant characteristics: including age, sex, BMI, foot posture [10], education, self-reported weightbearing hours, and weightbearing ankle dorsiflexion [11]. Participants were also asked their treatment preference and duration of symptoms.
(ii) Foot pain: measured using the Foot Health Status Questionnaire (FHSQ) pain subscale (primary outcome) [12], and a VAS to measure the severity of ‘first step’ and average pain.
(iii) Foot function: measured using the FHSQ foot function subscale [12].
(iv) Self-reported physical activity: measured using the 7-day Physical Activity Recall Questionnaire [13]. The results were converted to daily energy expenditure expressed in kcal/day [14].
(v) Fear-avoidance beliefs and feelings: measured using the Fear-Avoidance Components Scale (FACS) [15].
(vi) Plantar fascia thickness and hypoechogenicity: measured sonographically (at weeks 4 and 12) using a Siemens Acuson Antares (Siemens Healthcare GmbH, Erlangen, Germany) with a linear 5–12 MHz probe.

### Statistical analysis

Statistical analyses were performed using Stata version 16.0 (StataCorp LLC, Texas, United States of America). Standard tests of the distribution of continuous data were undertaken, and any variables with skewed data were transformed if appropriate. The normality of residuals was inspected with Kernel plots, P-P plots and Q-Q plots. Homoscedasticity was evaluated by plotting residuals versus predicted values and using the *imtest* and *hettest* commands in Stata. Multicollinearity was controlled by visually inspecting pairwise correlations and by ensuring the variation inflation factor was less than 10 for each predictor. Linearity was evaluated by plotting the standardised residuals against each of the predictor variables in the regression model using scatterplots and augmented partial residual plots [16]. Missing data were replaced using multiple imputation [17]. Little’s test was used to ensure data were missing completely at random (MCAR), and to determine the subsequent method for generating the imputed datasets [18]. Regression models were created using age, sex, BMI, and baseline values as predictors. The estimates from 30 imputed datasets were combined using Rubin’s rules [19]. We chose to use 30 imputed datasets to reduce sampling variability from the imputation process [17].

To identify predictor variables for inclusion in multivariable linear regression models, Pearson’s pairwise correlation coefficients were calculated between potential predictor variables and the dependent variables (the FHSQ foot pain subscale, the FHSQ foot function subscale, and first-step pain measured with a VAS). Significant pairwise correlation coefficients (*p* < 0.10) were entered in multiple linear regression models using forward hierarchical selection. Due to the sample size, only the strongest four predictor variables (determined by the magnitude of Pearson’s *r*) were entered into the model to prevent overfitting [20]. In order to predict response, baseline scores of the predictor variable were included in the model, as well as covariates age, sex and BMI. Separate regression models were developed for each intervention (i.e. foot orthoses and corticosteroid injection), dependent variable (i.e. foot pain, foot function and first-step pain), and at two timepoints (i.e. at weeks 4 and 12). Regression models were generated in Stata version 16.0 using the *mibeta* command, and the means of the imputed *R*^2^ estimates were transformed using Fisher’s *r* to *z* transformation (*fisherz* command) to provide a better estimate [21]. Significance for the multivariable regression models was α = 0.05.

## Results

A total of 220 people were screened for eligibility between May 2016 and June 2017, and 103 participants were randomised and received a treatment. Participant characteristics are outlined in Table 1. Data were missing for 6% (*n* = 7 participants) of the data at week 4, and 2% (*n* = 2 participants) of the data at week 12. The data were deemed to be missing completely at random (Little’s MCAR test: Chi-Square = 276.43, degrees of freedom 762, *p*-value > 1.00). The missing values were replaced using the predictive mean matching method of multiple imputation in Stata version 16.0.

**Table 1:** Participant characteristics and relevant baseline outcome measures

Characteristic	Foot orthoses (***n*** = 53)	Corticosteroid injection (***n*** = 50)
Age, years	42.9 (10.9)	44.9 (12.8)
Number of women, n (%)	33 (62.3)	30 (60.0)
Weight, kg	88.1 (21.5)	86.9 (21.7)
Height, m	1.7 (0.9)	1.7 (1.0)
Body mass index, kg/m^2^	31.1 (6.6)	29.7 (5.9)
Education, years	15.5 (2.7)	15.1 (3.6)
Duration of symptoms, months (median and interquartile range)	6 (8)	6 (8)
Weightbearing hours	6.9 (3.3)	7.2 (3.5)
Allocated to preferred treatment, n (%)	21 (39.6)	21 (42.0)
Plantar fascia thickness, mm^a^	5.9 (1.1)	5.8 (1.5)
Foot Posture Index^b^	3.6 (3.3)	3.7 (3.7)
Ankle dorsiflexion, degrees		
Knee extended	40.2 (6.9)	37.9 (6.9)
Knee flexed	45.9 (8.3)	44.7 (7.3)
**Baseline outcome measures**		
FHSQ pain subscale^c^	38.4 (17.3)	38.5 (17.0)
FHSQ footwear subscale^c^	43.1 (21.1)	50.3 (21.1)
Baseline pain (measured using a VAS)^d^	51.1 (16.7)	56.8 (17.9)
First-step pain (measured using a VAS)^d^	68.2 (14.9)	72.5 (16.4)
FACS^e^	30.8 (18.0)	29.6 (16.6)

*Abbreviations*: *FHSQ* Foot Health Status Questionnaire, *FACS* Fear-Avoidance Components Scale, *VAS* visual analogue scale

^a^Based on the most painful foot

^b^Scores range from − 12 (highly supinated foot) to + 12 (highly pronated foot). A score between 1 and 7 indicates a normal foot posture [22]

^c^Scores on the FHSQ range from 0 (most pain/difficulty with footwear) to 100 (no pain/no difficulty with footwear)

^d^Scores on the VAS range from 0 (no pain) to 100 (worst pain imaginable)

^e^Scores on the FACS range from 0 (no fear-avoidance beliefs) to 100 (extreme fear-avoidance beliefs)

### Univariable analyses

For foot orthoses, predictor variables that were significantly correlated (*p* < 0.10) with the dependent variables at weeks 4 and 12 are summarised in Table 2. For corticosteroid injection, predictor variables that were significantly correlated with the dependent variables at weeks 4 and 12 are summarised in Table 3. There were no significant predictors identified for foot pain and first step-pain at week 4.

**Table 2:** Significant predictor variables based on univariable analyses for foot orthoses at weeks 4 and 12

Dependent variable	Predictor variable	Pearson’s ***r***	***P***-value^a^
**Week 4**			
Foot pain	Ankle dorsiflexion – knee extended	0.36	0.013
	Foot Posture Index	−0.35	0.015
Foot function	FACS	−0.63	0.001
	FHSQ footwear subscale	0.37	0.010
First-step pain	Ankle dorsiflexion – knee extended	−0.39	0.006
	Preference for foot orthoses	0.28	0.059
**Week 12**			
Foot pain	BMI	−0.43	0.001
	Duration of symptoms	−0.28	0.047
Foot function	BMI	−0.46	0.000
	FACS	−0.38	0.005
	FHSQ footwear subscale	−0.36	0.009
	First-step pain	−0.25	0.077
First-step pain	BMI	0.43	0.002
	Weightbearing hours	0.29	0.031
	Age	−0.29	0.036

*Abbreviations*: *FACS* Fear-Avoidance Components Scale, *FHSQ* Foot Health Status Questionnaire, *BMI* body mass index

^a^The alpha level to include predictors was 0.10

**Table 3:** Significant predictors based on univariable analyses for corticosteroid injection at weeks 4 and 12

Dependent variable	Predictor variable	Pearson’s ***r***	***P***-value^a^
**Week 4**^**b**^			
Foot function	FHSQ footwear subscale	−0.45	0.001
	Baseline pain (measured using a VAS)	−0.32	0.021
	FACS	−0.31	0.031
**Week 12**			
Foot pain	Weightbearing hours	−0.33	0.020
Foot function	FACS	−0.42	0.002
	Baseline pain (measured using a VAS)	−0.39	0.005
	FHSQ footwear subscale	−0.29	0.043
	First-step pain	−0.24	0.094
First-step pain	Weightbearing hours	0.34	0.017

*Abbreviations*: *FHSQ* Foot Health Status Questionnaire, *FACS* Fear-Avoidance Components Scale, *VAS* visual analogue scale

^a^The alpha level to include predictors was 0.10

^b^There were no significant predictors at week 4 for foot pain and first-step pain

### Multivariable linear regression analyses

#### Foot orthoses

Table 4 displays the results of the multivariable linear regression analyses for the dependant variables foot pain, foot function, and first-step pain at weeks 4 and 12. For foot pain at week 4, a model that included ankle dorsiflexion with the knee extended explained 16% of the variance in the FHSQ foot pain subscale (F_5, 44.3_ = 2.66, *p* = 0.034). Univariable analysis found that the Foot Posture Index was a significant predictor, however it was not significant in the multivariable model. This model suggests that greater ankle dorsiflexion predicted reduction in foot pain at week 4. For foot function at week 4, a model that included the FACS explained 43% of the variance in the FHSQ foot function subscale (F_5, 43.2_ = 6.89, *p* = 0.001). Univariable analysis found the FHSQ footwear subscale was a significant predictor, however it was not significant in the multivariable model. This model suggests that lower fear-avoidance predicted improvement in foot function at week 4. For first-step pain at week 4, a model that included ankle dorsiflexion with the knee extended explained 15% of the variance using a VAS, however it was not statistically significant (F_5, 43.8_ = 2.26, *p* = 0.064). Univariable analysis found participant preference to receive foot orthoses was a significant predictor, however it was not significant in the multivariable model.

**Table 4:** Multivariable linear regression models for foot orthoses at weeks 4 and 12

Dependent variable		Unstandardised coefficients		Standardised coefficients				
	Model^a^	β	SE β	β	***t***-value	***P***-value^**b**^	***R***^**2**^	Adjusted ***R***^**2**^
**Week 4**								
Foot pain	Ankle dorsiflexion – knee extended	0.813	0.377	0.309	2.15	0.037	0.25	0.16*
Foot function	FACS	−0.271	0.132	−0.318	−2.06	0.047	0.49	0.43*
First-step pain	Ankle dorsiflexion – knee extended	−1.327	0.529	−0.353	−2.51	0.016	0.24	0.15
**Week 12**								
Foot pain	BMI	−1.237	0.424	−0.389	−2.92	0.006	0.39	0.33*
	Duration of symptoms	−0.364	0.175	−0.251	−2.08	0.044		
Foot function	BMI	−0.671	0.288	−0.298	−2.33	0.025	0.42	0.37*
First-step pain	BMI	1.220	0.430	0.406	2.83	0.007	0.25	0.19*
	Age	−0.503	0.228	−0.276	−2.20	0.033		

*Abbreviations*: *SE* standard error, *FACS* Fear-Avoidance Components Scale, *BMI* body mass index

^a^Adjusted for age, sex, BMI, and baseline scores of the dependent variable

^b^The *p*-value represents the statistical significance of individual variables

* The *p-*value of the adjusted *R*^2^ was < 0.05

For foot pain at week 12, a model that included BMI and duration of symptoms explained 33% of the variance in the FHSQ foot pain subscale (F_5, 44.6_ = 5.56, *p* < 0.001). This model suggests that lower BMI and a shorter duration of symptoms predicted reduction in foot pain at week 12. For foot function at week 12, BMI explained 37% of the variance in the FHSQ foot function subscale (F_4, 45.3_ = 7.68, *p* < 0.001). Univariable analysis found fear avoidance, the FHSQ footwear subscale, and first-step pain were significant predictors, however they were not significant in the multivariable model. This model suggests that lower BMI predicted improvement in foot function at week 12. For first-step pain at week 12, a model containing age and BMI explained 19% of the variance in a VAS (F_4, 45.5_ = 3.68, *p* = 0.011). This model suggests that lower BMI and older age predicted reduction in first-step pain at week 12. Univariable analysis found weightbearing hours was a significant predictor, however it was not significant in the multivariable model.

#### Corticosteroid injection

Table 5 displays the results of the multivariable linear regression analyses at week 12 (there were no significant predictors in the multivariable models at week 4). For foot pain at week 12, a model that included weightbearing hours explained 25% of the variance in FHSQ foot pain (F_5, 42_ = 4.10, *p* = 0.004). This model suggests that less weightbearing hours predicted reduction in foot pain at week 12. For foot function at week 12, a model that included baseline pain measured using a VAS explained 38% of the variance in the FHSQ foot function subscale (F_5, 41.8_ = 6.52, *p* < 0.001). This model suggests that lower baseline foot pain predicted improvement in foot function at week 12. Univariable analysis found that the FACS, the FHSQ footwear subscale and first-step pain were significant predictors, however they were not significant in the multivariable model. For first-step pain at week 12, a model that included weightbearing hours explained 10% of the variance in a VAS (F_5, 42.0_ = 1.99, *p* = 0.100), however it was not statistically significant.

**Table 5:** Multivariable linear regression models for corticosteroid injection at weeks 4 and 12

		Unstandardised coefficients		Standardised coefficients				
Dependent variable^a^	Model^b^	β	SE β	β	***t***-value	***P***-value^**c**^	***R***^**2**^	Adjusted ***R***^**2**^
**Week 12**								
Foot pain	Weightbearing hours	−1.742	1.011	−0.235	−1.72	0.092	0.32	0.25*
Foot function	Baseline pain (measured using a VAS)	−0.323	0.156	−0.285	−2.07	0.046	0.44	0.38*
First-step pain	Weightbearing hours	2.482	1.339	0.284	7.85	0.071	0.19	0.10

*Abbreviations*: *SE* standard error, *BMI* body mass index, *VAS* visual analogue scale

^a^There were no significant predictors at week 4

^b^Adjusted for age, sex, BMI, and baseline scores of the dependent variable

^c^The *p*-value represents the statistical significance of individual variables

* The *p-*value of the adjusted *R*^2^ was < 0.05

## Discussion

This aim of this study was to identify variables from a recent randomised trial that predict response to foot orthoses and corticosteroid injection in people with plantar heel pain [6]. For each intervention, we evaluated whether baseline variables (age, weight, height, BMI, sex, education, foot pain, foot function, fear-avoidance beliefs and feelings, foot posture, ankle dorsiflexion, plantar fascia thickness, and treatment preference) could be used to predict change of foot pain, foot function, and first-step pain. We found that predictors differed for each intervention, dependent variable, and at each timepoint (i.e. at 4 weeks and 12 weeks).

### Foot orthoses

At week 4, *greater* ankle dorsiflexion predicted reduction in foot pain for people who received foot orthoses. Previous studies have found that *reduced* ankle dorsiflexion is associated with plantar heel pain [23–27], while other studies have found that *greater* ankle dorsiflexion is associated with plantar heel pain [28, 29]. One past study that evaluated predictors of response to foot orthoses found that participants with *markedly* reduced ankle dorsiflexion had a better response [7]. However, the statistical analysis used in this study is quite different to our study, which makes a direct comparison difficult. Further research will be required to understand whether ankle dorsiflexion can be used to predict response for people who receive foot orthoses for plantar heel pain.

Univariable analyses found that the FACS was associated with foot function for both interventions and at both timepoints. This finding is supported by a past study that found a similar pain-related fear construct, fear of movement/(re) injury, was associated with worse foot function in people with plantar heel pain [30]. However, using multivariable analysis in our study, lower fear-avoidance was a *predictor* of improvement in foot function for the foot orthoses group at week 4 only; explaining 43% of the variance in the FHSQ foot function subscale. These findings suggest that fear-avoidance beliefs may be *associated* with worse foot function, however they only predict worse foot function in the short term with the use of foot orthoses. The significance of these findings is uncertain, however they may help to explain why some patients do not respond to foot orthoses in the short term. Indeed, past research has found that fear-avoidance related symptoms negatively affect treatment outcomes for patients with chronic musculoskeletal pain [31–33]. Therefore, patients with elevated fear-avoidance beliefs and feelings may benefit from additional interventions to address these beliefs.

At week 12, lower BMI was a consistent predictor of a favourable response to foot orthoses. Lower BMI predicted reduction in foot pain (in conjunction with shorter duration of symptoms), improvement in foot function, and reduction in first-step pain (in conjunction with younger age). A past meta-analysis identified BMI as one of the most important factors associated with plantar heel pain, although this finding differs between athletic and non-athletic populations [34].

### Corticosteroid injection

At week 4, there were no significant predictors for foot pain (including first-step pain) and the significant predictors for foot function identified by univariable analyses were not significant in multivariable models. Difficulty identifying predictors of response following corticosteroid injection is not unique to plantar heel pain, and has also been reported for other conditions such as knee [35] and hip osteoarthritis [36], and idiopathic trigger finger [37]. For plantar heel pain, this is the first study to predict change in pain or function following a corticosteroid injection, with the only previous study predicting change in tenderness thresholds [38].

At week 12, less baseline weightbearing hours predicted reduction in foot pain and lower baseline foot pain (i.e. foot pain severity measured using a VAS) predicted improvement in foot function. A previous meta-analysis that included four studies found that more weightbearing hours was associated with plantar heel pain [34]. Our study is the first to identify that this variable can *predict* response in those with plantar heel pain, although it is unclear why this variable was only a predictor for corticosteroid injection. Foot orthoses can modify plantar pressures at the heel [39–42], which may explain why weightbearing hours was only a predictor for corticosteroid injection.

For foot function, lower baseline pain predicted improvement in foot function and explained 38% of the variance in this model. There have been contrasting findings from past studies regarding the association of foot pain on function. One study that evaluated the relationship between pain intensity and function in those with plantar heel pain found no association [43]. However, research with older adults from the community has found that foot pain is associated with functional impairment [44, 45]. Our study suggests that baseline foot pain is important for predicting improvement in foot function, however this finding is only apparent following corticosteroid injection.

### Limitations

The findings of this study should be interpreted with respect to some limitations. First, the data for this study is a secondary analysis of a randomised trial that evaluated foot orthoses and corticosteroid injection for plantar heel pain. Therefore, the data obtained from participants relates to the aim of the randomised trial rather than the aim of this study, which may have influenced the data collected. Second, the sample size was determined to power the randomised trial rather than this study, which limited the number of predictors that could be included in the multivariable regression models. Third, the results of this study are specific to the interventions used in the randomised trial. It is possible that we could have obtained different findings if a different type of orthosis or corticosteroid injection was used. Finally, the overall variance explained by the models is modest and there may be important predictors that were not included as outcome measures in the randomised trial. Therefore, important predictors of response may not have been measured, such as comorbidities or certain imaging features.

### Future research

There are no studies that have performed an a priori evaluation of the predictors of response to treatment of plantar heel pain. An a priori evaluation can overcome many of the limitations discussed above and generate more robust findings. Understanding variables that predict response is important because it can help health professionals choose more appropriate interventions for their patients and can assist with the design of clinically relevant randomised trials. For example, the findings of this study may inform randomised trials of foot orthoses that may stratify by predictors, such as ankle dorsiflexion. Another area for future research is that we found no predictors of response for participants who received a corticosteroid injection in the short term. Change in pain is important for patients with plantar heel pain and future research is needed to identify important predictors to improve outcome for corticosteroid injection [46].

## Conclusions

For individuals with plantar heel pain, the predictors of response differed for foot orthoses and corticosteroid injection. For foot orthoses, we found that greater ankle dorsiflexion and lower BMI predicted reduction in foot pain, while lower fear-avoidance beliefs and feelings, as well as lower BMI predicted improvement in foot function. For corticosteroid injection, we found that less weightbearing hours predicted reduction in foot pain, and lower baseline foot pain predicted improvement in foot function. These findings may be used to inform the design of future research.

## Data Availability

Data and accompanying material are available from the lead author (GAW) upon reasonable request.

## Abbreviations

VAS: Visual analogue scale
BMI: Body mass index
FHSQ: Foot Health Status Questionnaire
FACS: Fear-Avoidance Components Scale

## References

[1] Irving DB, Cook JL, Young MA, Menz HB (2008). Impact of chronic plantar heel pain on health-related quality of life. J Am Podiatr Med Assoc.

[2] Thomas MJ, Whittle R, Menz HB, Rathod-Mistry T, Marshall M, Roddy E (2019). Plantar heel pain in middle-aged and older adults: population prevalence, associations with health status and lifestyle factors, and frequency of healthcare use. BMC Musculoskelet Disord.

[3] Hill CL, Gill TK, Menz HB, Taylor AW. Prevalence and correlates of foot pain in a population-based study: the North West Adelaide Health Study. J Foot Ankle Res. 2008;1:2. 10.1186/1757-1146-1-2 PMC2547889 18822153

[4] Dunn JE, Link CL, Felson DT, Crincoli MG, Keysor JJ, McKinlay JB (2004). Prevalence of foot and ankle conditions in a multiethnic community sample of older adults. Am J Epidemiol.

[5] Lopes AD, Hespanhol LC, Yeung SS, Costa LOP (2012). What are the main running-related musculoskeletal injuries?. Sport Med.

[6] Whittaker GA, Munteanu SE, Menz HB, Gerrard JM, Elzarka A, Landorf KB (2019). Effectiveness of foot orthoses versus corticosteroid injection for plantar heel pain: the SOOTHE randomized clinical trial. J Orthop Sport Phys Ther.

[7] Wrobel JS, Fleischer AE, Matzkin-Bridger J, Fascione J, Crews RT, Bruning N (2016). Physical examination variables predict response to conservative treatment of nonchronic plantar fasciitis: secondary analysis of a randomized, placebo-controlled footwear study. PM&R..

[8] Whittaker GA, Munteanu SE, Menz HB, Elzarka A, Landorf KB (2017). Corticosteroid injections compared to foot orthoses for plantar heel pain: protocol for the SOOTHE heel pain randomised trial. Contemp Clin Trials Commun.

[9] Martin RL, Davenport TE, Reischl SF, McPoil TG, Matheson JW, Wukich DK (2014). Heel pain—plantar fasciitis: revision 2014. J Orthop Sport Phys Ther..

[10] Redmond AC, Crosbie J, Ouvrier RA. Development and validation of a novel rating system for scoring standing foot posture: the Foot Posture Index. Clin Biomech (Bristol Avon). 2006;21:89–98. 10.1016/j.clinbiomech.2005.08.002 16182419

[11] Munteanu SE, Strawhorn AB, Landorf KB, Bird AR, Murley GS (2009). A weightbearing technique for the measurement of ankle joint dorsiflexion with the knee extended is reliable. J Sci Med Sport.

[12] Bennett P, Patterson C, Wearing S, Baglioni T (1998). Development and validation of a questionnaire designed to measure foot-health status. J Am Podiatr Med Assoc.

[13] Sallis JF, Haskell WL, Wood PD, Fortmann SP, Rogers T, Blair SN (1985). Physical activity assessment methodology in the Five-City project. Am J Epidemiol.

[14] Richardson M, Ainsworth BE, Jacobs DR, Leon AS. Validation of the Stanford 7-day Recall to assess habitual physical activity. Ann Epidemiol. 2001;11:145–53. 10.1016/s1047-2797(00)00190-3 11164131

[15] Neblett R, Mayer TG, Hartzell MM, Williams MJ, Gatchel RJ. The Fear-Avoidance Components Scale (FACS): development and psychometric evaluation of a new measure of pain-related fear avoidance. Pain Pract. 2016;16:435–50. 10.1111/papr.12333 26228238

[16] Chen X, Ender P, Mitchell M, Wells C (2003). Chapter 2 - regression diagnostics. Regression with Stata.

[17] Sterne JAC, White IR, Carlin JB, Spratt M, Royston P, Kenward MG (2009). Multiple imputation for missing data in epidemiological and clinical research: potential and pitfalls. BMJ..

[18] Little RJA (1988). A test of missing completely at random for multivariate data with missing values. J Am Stat Assoc.

[19] Rubin DB (1987). Multiple imputation for nonresponse in surveys.

[20] Wilson Vanvoorhis CR, Morgan BL (2007). Understanding power and rules of thumb for determining sample sizes. Tutor Quant Methods Psychol.

[21] Harel O (2009). The estimation of *R*^2^ and adjusted *R*^2^ in incomplete data sets using multiple imputation. J Appl Stat.

[22] Redmond AC, Crane YZ, Menz HB. Normative values for the Foot Posture Index. J Foot Ankle Res. 2008;1:6. 10.1186/1757-1146-1-6 PMC2553778 18822155

[23] Kibler W, Ben GC, Chandler TJ (1991). Functional biomechanical deficits in running athletes with plantar fasciitis. Am J Sports Med.

[24] Sullivan J, Burns J, Adams R, Pappas E, Crosbie J (2015). Musculoskeletal and activity-related factors associated with plantar heel pain. Foot Ankle Int..

[25] Riddle DL, Pulisic M, Pidcoe P, Johnson RE. Risk factors for plantar fasciitis: a matched case-control study. J Bone Jt Surg Am. 2003;85:872–7. 10.2106/00004623-200305000-00015 12728038

[26] Labovitz JM, Yu J, Kim C (2011). The role of hamstring tightness in plantar fasciitis. Foot Ankle Spec.

[27] Bolívar YA, Munuera PV, Padillo JP (2013). Relationship between tightness of the posterior muscles of the lower limb and plantar fasciitis. Foot Ankle Int..

[28] Pohl MB, Hamill J, Davis IS (2009). Biomechanical and anatomic factors associated with a history of plantar fasciitis in female runners. Clin J Sport Med.

[29] Irving DB, Cook JL, Young MA, Menz HB (2007). Obesity and pronated foot type may increase the risk of chronic plantar heel pain: a matched case-control study. BMC Musculoskelet Disord.

[30] Cotchett M, Lennecke A, Medica VG, Whittaker GA, Bonanno DR (2017). The association between pain catastrophising and kinesiophobia with pain and function in people with plantar heel pain. Foot (Edinb).

[31] Bergbom S, Boersma K, Linton SJ (2012). Both early and late changes in psychological variables relate to treatment outcome for musculoskeletal pain patients at risk for disability. Behav Res Ther.

[32] Denison E, Åsenlöf P, Sandborgh M, Lindberg P (2007). Musculoskeletal pain in primary health care: subgroups based on pain intensity, disability, self-efficacy, and fear-avoidance variables. J Pain.

[33] Wertli MM, Rasmussen-Barr E, Held U, Weiser S, Bachmann LM, Brunner F (2014). Fear-avoidance beliefs—a moderator of treatment efficacy in patients with low back pain: a systematic review. Spine J.

[34] van Leeuwen KDB, Rogers J, Winzenberg T, van Middelkoop M (2016). Higher body mass index is associated with plantar fasciopathy/‘plantar fasciitis’: systematic review and meta-analysis of various clinical and imaging risk factors. Br J Sports Med.

[35] Maricar N, Callaghan MJ, Felson DT, O’Neill TW (2013). Predictors of response to intra-articular steroid injections in knee osteoarthritis: a systematic review. Rheumatology..

[36] Hirsch G, Kitas G, Klocke R (2013). Intra-articular corticosteroid injection in osteoarthritis of the knee and hip: factors predicting pain relief—a systematic review. Semin Arthritis Rheum.

[37] Julka A, Vranceanu AM, Shah AS, Peters F, Ring D (2012). Predictors of pain during and the day after corticosteroid injection for idiopathic trigger finger. J Hand Surg Am.

[38] Chen C-M, Hsu H-C, Tsai W-C, Chang C-H, Lin C-H, Chen K-H, et al. The “bodily pain” scale of the Short Form-36 questionnaire is a predictor of outcome in patients who receive ultrasound-guided corticosteroid injection for plantar fasciitis—a preliminary study. J Musculoskelet Pain. 2014;22:335–40.

[39] Bonanno DR, Landorf KB, Menz HB (2011). Pressure-relieving properties of various shoe inserts in older people with plantar heel pain. Gait Posture.

[40] Burns J, Wegener C, Begg L, Vicaretti M, Fletcher J (2009). Randomized trial of custom orthoses and footwear on foot pain and plantar pressure in diabetic peripheral arterial disease. Diabet Med.

[41] Najafi B, Barnica E, Wrobel JS, Burns J (2012). Dynamic plantar loading index: understanding the benefit of custom foot orthoses for painful pes cavus. J Biomech.

[42] Redmond A, Lumb PS, Landorf K (2000). Effect of cast and noncast foot orthoses on plantar pressure and force during normal gait. J Am Podiatr Med Assoc.

[43] Riddle DL, Pulisic M, Sparrow K (2004). Impact of demographic and impairment-related variables on disability associated with plantar fasciitis. Foot Ankle Int.

[44] Menz HB, Lord SR (2001). Foot pain impairs balance and functional ability in community-dwelling older people. J Am Podiatr Med Assoc.

[45] Mickle KJ, Munro BJ, Lord SR, Menz HB, Steele JR (2011). Cross-sectional analysis of foot function, functional ability, and health-related quality of life in older people with disabling foot pain. Arthritis Care Res (Hoboken).

[46] Cotchett M, Rathleff MS, Dilnot M, Landorf KB, Morrissey D, Barton C (2020). Lived experience and attitudes of people with plantar heel pain: a qualitative exploration. J Foot Ankle Res..

